# Validation of a Model Estimating the Budget Impact of Video Capsule Endoscopy for Surveillance of Crohn’s Disease in an Italian Center

**DOI:** 10.36469/001c.92880

**Published:** 2024-03-07

**Authors:** Rhodri Saunders, Carlo Calabrese, Dania Gelli, Jason Davis, Rafael Torrejon Torres

**Affiliations:** 1 Coreva Scientific GmbH, Königswinter, Germany; 2 IBD Unit IRCCS, Azienda Ospedaliero-Universitaria di Bologna, Bologna, Italy; 3 Università di Bologna, Bologna, Italy

**Keywords:** surgery, biologics, symptomatology, remission, Crohn’s disease management, capsule endoscopy

## Abstract

**Background:** Crohn’s disease is a chronic ailment affecting the gastrointestinal tract. Mucosal healing, a marker of reduced disease activity, is currently assessed in the colonic sections using ileocolonoscopy and magnetic resonance enteroscopy. Video capsule endoscopy (VCE) offers visualization of the entire GI mucosae.

**Objective:** To validate a Crohn’s disease model estimating the budget impact of VCE compared with the standard of care (SOC) in Italy.

**Methods:** A patient-level, discrete-event simulation was developed to estimate the budget impact of VCE compared with SOC for Crohn’s disease surveillance over 5 years in the Italian setting. Input data were sourced from a physician-initiated study from Sant’Orsola-Malpighi Hospital in Bologna, Italy, and the literature. The care pathway followed hospital clinical practice. Comparators were the current SOC (ileocolonoscopy, with or without magnetic resonance enteroscopy) and VCE. Sensitivity analysis was performed using 500-patient bootstraps. A comparative analysis regarding clinical outcomes (biologics use, surgical interventions, symptom remission) was performed to explore the validity of the model compared with real-world data. Cumulative event incidences were compared annually and semi-annually. Bayesian statistical analysis further validated the model.

**Results:** Implementing VCE yielded an estimated €67 savings per patient per year, with savings in over 55% of patients, compared with SOC. While annual costs are higher up to the second year, VCE becomes cost saving from the third year onward. The real-world validation analysis proved a good agreement between the model and real-world patient records. The highest agreement was found for biologics, where Bayesian analysis estimated an 80.4% probability (95% CI: 72.2%-87.5%) that a decision maker would accept the result as an actual reflection of real-world data. Even where trend data diverged (eg, for surgery [43.1% likelihood of acceptance, 95% CI: 33.7%-52.8%]), the cumulative surgery count over 5 years was within the margin of error of the real-world data.

**Conclusions:** Implementing VCE in the surveillance of patients with Crohn’s disease and small bowel involvement may be cost saving in Italy. The congruence between model predictions and real-world patient records supports using this discrete-event simulation to inform healthcare decisions.

## BACKGROUND

Crohn’s disease (CD) is a chronic, relapsing, and heterogeneous inflammatory disorder of the gastrointestinal (GI) tract, most often diagnosed in the terminal ileum and the colon.^1–4^ The standard of care (SOC) for diagnosing and monitoring CD is a combination of ileocolonoscopy (ILE), which allows for direct visualization and biopsy of the lower GI mucosa, and magnetic resonance enteroscopy (MRE) for the indirect assessment in the small bowel and upper GI tract.^3,5^ Video capsule endoscopy (VCE) has emerged as a versatile tool for noninvasive diagnosis and monitoring of CD throughout the small intestine,^2,6^ with the same sensitivity as SOC in the lower GI tract^7^ and better specificity than MRE in the small bowel.^8,9^ VCE also provides an accurate assessment of mucosal healing, a widely accepted marker of remission,^3,10,11^ and better patient acceptance.^12–14^ VCE may allow for a readier and more adaptable therapeutic follow-up, particularly in the medium- to long-term management of small bowel lesions,^7^ where it showed the potential to optimize the management of CD.^15^

VCE has garnered attention as a minimally invasive diagnostic in various settings, particularly for evaluating and monitoring CD.^16–20^ However, VCE’s health-economic implications in CD surveillance remain understudied,^21^ without evidence specific to Italian healthcare. We had previously shown VCE as a cost-effective alternative in the mid-to-long term in the United Kingdom and United States, with higher initial costs recouped through improved management over time, using a patient-level discrete-event simulation model.^22,23^ However, the model lacks external validation for its accuracy against real-world data. Therefore, this work leverages published data from Calabrese et al,^15^ adapting the model to the local specifics and estimating the potential budget impact of adopting VCE compared with the SOC (ILE ± MRE) for CD surveillance in Italy. To determine the validity of this model, we assessed the model’s precision on clinical outcomes relevant to resource use and management of CD by comparing the generated longitudinal patient and real-world patient data, per recommendations from the International Society for Pharmacoeconomics and Outcomes Research (ISPOR) and Bayesian statistical analysis.^24^

## METHODS

### Analytic Approach

**Figure 1.**
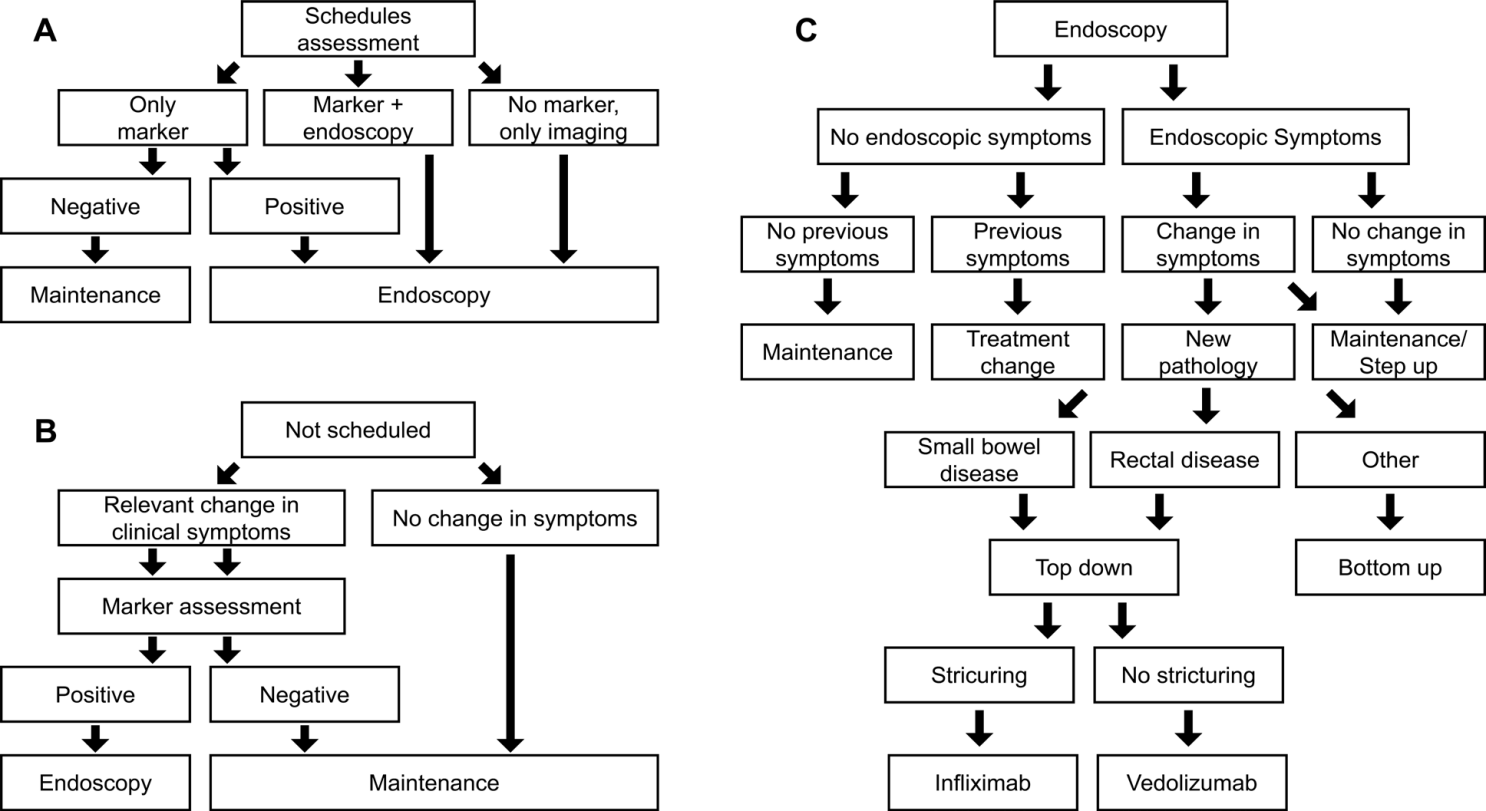
Diagnostic Workflow of the Model Patients can be eligible for endoscopic monitoring in each cycle by being regularly scheduled for monitoring (**A**) or scheduled for monitoring due to disease flare suspicion (**B**). In both scenarios, a marker test is conducted to confirm disease activity. Patients not scheduled for regular maintenance endoscopy get scheduled for endoscopy upon a positive result of the marker testing. Diagnosed complications result in surgery.

A discrete-event, patient-level simulation (**Figure 1**) with an underlying semi-Markov model, as described in previous publications,^22,23^ was adapted to the Italian setting based on expert clinician inputs and available guidelines.^3,25–27^ The model compares expected outcomes using VCE in CD surveillance in place of SOC (ILE ± MRE) by simulating CD progression based on individual patient characteristics, sensitivity and specificity of diagnostics (VCE or SOC), and treatment efficacy. The model was utilized to calculate the budget impact of introducing VCE in surveillance of patients diagnosed with small bowel CD compared with SOC surveillance. From the publication by Calabrese et al, data on 276 patients with CD were available for the model.^15^ Each individual-level patient record was replicated 10 times to provide a 2760-simulation set to better account for potential uncertainty.

**Model overview:** The simulation spans 4.5 years of follow-up, using each patient’s first collected data point as a baseline input for the model. Over time, patient characteristics such as age, disease status, clinical disease activity index (CDAI), structuring, and abscess are updated. Age is simply updated in yearly increments, whereas CD-related characteristics are updated each cycle based on an underlying model with, for example, a semi-Markov model used to incorporate CD symptom progression. Each model cycle simulates a period of 3 months. In each cycle, each patient is evaluated for whether there is a scheduled physician visit. If so, then the patient undergoes planned surveillance using one of three options depending on their disease status and symptomatology (**Figure 1A**). If there was no scheduled physician visit, the patient is assessed for changes in CD symptomatology (eg, flares) that could signal an unplanned physician visit. If these are present, marker assessment with fecal calprotectin (fCal) is performed; otherwise, the patient remains on current maintenance (**Figure 1B**). Following either approach, patients with a positive market test or scheduled for endoscopy receive endoscopy as either SOC or VCE (depending on the arm of the model) and follow the procedure in **Figure 1C** to determine a diagnosis and treatment option as required.

Next to the diagnostic workflow of the model, symptom progression is estimated in the underlying semi-Markov process with monthly transitions (**Tables S1 and S2**); therefore, as a cycle is 3 months in length, multiple disease escalations or regressions per cycle are possible. For example, a patient with mild CD could have a flare in month 1 (of 3), have this resolve in month 2 (of 3), and then enter remission in month 3 (of 3). They would, therefore, in the model follow-up, go from mild CD to remission. Similarly, the same patient could have no change in months 1 and 2 (of 3) and a flare in month 3 (of 3), entering the next model cycle with moderate CD. If mild, moderate, or severe CD persists at the subsequent follow-up then a change in treatment, using either a bottom-up or top-down approach is considered (**Figure 1C**). The treatment is adjusted if a new pathology has developed (eg, rectal disease or small bowel presentation). Furthermore, the patient is directed to surgery if a complication is diagnosed.

The main model outcomes are the initiation of biologic treatment, biological treatment duration, and evolution of biological utilization through the model time frame. The secondary model’s outcomes are the incidence of surgery and remission. Using the Crohn’s Activity Index as a proxy of symptomatology, remission was defined as an asymptomatic period (CDAI score <150) immediately following active disease (CDAI score ≥150). Staggered degrees of symptomatology and underlying active disease were mild (150 ≤ CDAI < 220), moderate (220 ≤ CDAI < 450), and severe (CDAI ≥ 450).

**Model input data**: For the baseline set of patients, real-world demographic and clinical outcome data in terms of biologics use, surgery occurrence, and the cumulative incidence of remission and flares were sourced from Calabrese et al,^15^ a monocentric, matched-cohorts, retrospective, physician-initiated study. It investigated the impact of capsule endoscopy compared to the SOC in patients with CD at the Sant’Orsola-Malpighi Hospital in Bologna, Italy, (IRCCS Azienda Ospedaliero-Universitaria di Bologna–Policlinico di Sant’Orsola) between 1999 and 2019.^15^ In Calabrese et al, entry into the database prior to 1999, no diagnosed disease location, no initial CDAI, use of VCE in addition to ILE/MRE, and more than 2 consecutive missing data points (1 year) constituted exclusion criteria. Patients with the follow-up data in 6-month intervals up to 5 years after database entry were selected.

Anonymized patient records retained served uniquely as inputs for the model to generate representative cohorts for the two simulation arms. Event incidences and costs were identified in a structured literature review and hand searches. This included data on adverse events and symptomatology, such as subsequent hospitalization, bowel obstruction, GI bleeding, and infection following a capsule retention. Key inputs for each surveillance modality are presented in **Table 1**. Patient characteristics such as age, time from diagnosis, and time on treatment are updated according to the individual-level procession through the model. Costs were obtained from the 2020 IT-DRG scheme for Emilia Romagna (**Table S3**).

**Table 1.** Efficacy, Safety, and Cost Data by Surveillance Modality

	**fCal Test**	**VCE**	**Ileocolonoscopy**	**MRE**
Sensitivity	78.8^13^	83%^9^ SB: 97%^9^	91%^9^ SB: Not used	71%^9^
Specificity	97.2^13^	88%^9^ SB: 87%^9^	89%^9^ SB: Not used	66%^9^
Subsequent hospitalization	0%	0%	1.63%^14^	0%
Bowel obstruction	0%	0%	0.08%^15^	0%
GI bleeding	0%	0%	0.42%^16^	0%
Infection	0%	0%	4%^17^	
Capsule retention				
With patency capsule	0%	0%^a^	0%	0%
Complete procedures	100%^b^	88.7%^18^	86.9%^19^	100%^b^
Cost per procedure, € (DRG code used)	12,05 (90.12.A Veneto)^28^	850.00 (45.13.1)^29^	74.00 (45.23)^29^	160.10 (88.95.4)^29^

Together, ileocolonoscopy and MRE form the standard of care.

^a^Based on medical records, assuming the use of patency capsule.

^b^Assumed to be always completed.

Abbreviations: fCal, fecal calprotectin; MRE, magnetic resonance enterography; SB, small bowel; VCE, video capsule endoscopy.

**Sensitivity analysis:** One thousand populations, 500 patients each, were bootstrapped to test the robustness of results in a multivariate sensitivity analysis.^30^ Median and interquartile ranges were estimated on bootstrapped populations. Only patients who had changed management practices were considered in the analysis, with single capsule endoscopy being a criterion during the model time horizon. A ±20% change in cost inputs was used in the one-way sensitivity analysis.

### Real-World Validation

An analysis was conducted to explore the external validity of the previously described model comparing the model outcomes with real-world data. To account for uncertainty, each patient was simulated 100 times during validation to account for random sampling and real-life treatment pathway uncertainty. Two outcome definitions (permissive and restrictive) influencing the determination of the compared clinical outcomes, as outlined in **Table S4**, were used for the analysis. For example, in the permissive definition any 6-month period with a CDAI ≤150 was considered remission, whereas in the restrictive definition this was extended to require a 12-month period with CDAI ≤150.

Under these definitions, and using the 276 baseline patient records, 27 600 simulated patients were to be funneled through the model. Clinical outcomes were compared between the model and real-world data. The cumulative incidence and the disease burden of clinical outcomes in terms of resource use (use of biologics and number of surgeries) and the course of disease (duration of remission) were compared to explore the goodness of the model.

### Bayesian Validation

The model was validated for its accuracy in predicting biologics treatment, surgeries, and remission using the Bayesian approach that Corro Ramos and colleagues proposed.^31^ The method assumes a 50% probability that a hypothetical decision maker would accept the model results as an accurate representation of the true value. The model’s success rate in predicting the empirical outcome is then determined to calculate the likelihood that the decision maker would accept the results, starting from the initial assumption of 50% acceptance. The model validity was computed as the likelihood of acceptance based on the proximity to the empirical value (ie, the model output is considered valid if within the uncertainty interval of the real value ≥50% of the time). Validity percentages were calculated at 1%, 5%, 10%, and 20% proximity. The validity analysis was also conducted, assuming a target acceptance rate of 80%. As cumulative data is unsuitable for competing-risk analyses and does not fulfill the proportional hazard requirement for survival analysis, hypothesis tests were not performed. Nonetheless, cumulative series are presented for visual comparison.

### Data Segregation

The discrete-event simulation used peer-reviewed published literature for all outcome events and transition probabilities. R.T.T. and R.S. developed and adapted the model to the Italian setting. No real-world patient data were used during the model development phase.

## RESULTS

### Model Results and Cost Calculations

We gauged the budget impact of implementing VCE in monitoring patients with small bowel CD diagnoses compared with using exclusively SOC (base case) in the validated model. To account for uncertainty, each patient was reproduced 10 times to provide an overall population of 2760. The model estimated mean annual costs of €6047 per patient in the base case vs €5980 per patient in the VCE scenario. VCE in the surveillance of patients with CD and small bowel involvement saved, on average, €67 per patient per year, or €221 by year 5.

**Figure 2.**
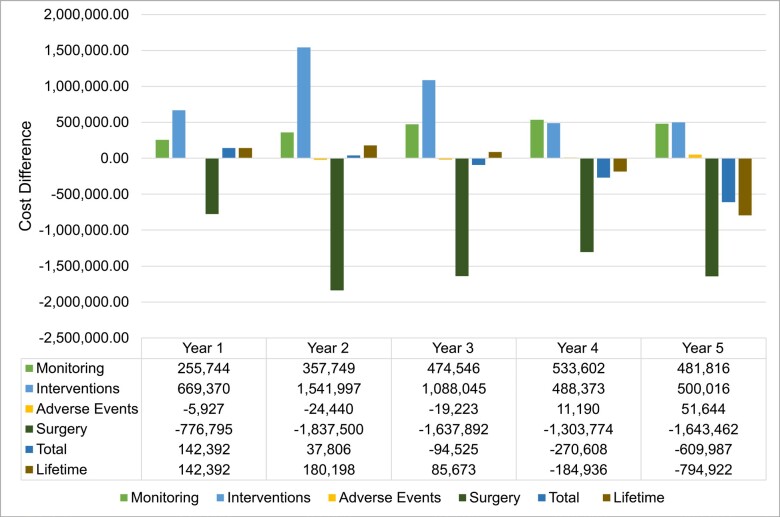
Cost Difference Breakdown by Year of Implementing VCE vs SOC Cost differences per groups (monitoring, interventions, adverse events, surgery) adding up to the total cost difference per year as well as accumulating cost differences over the 5-year time horizon.

Overall, VCE implementation incurred higher annual costs until the second year; the trend reversed from year 3, leading to cost savings from year 4 (**Figure 2**). A shifting balance between reduced surgery costs and increased monitoring and intervention costs drives this progression. Adverse event cost contribution is negligible.

**Sensitivity analysis:** Multiparameter sensitivity analysis was performed by comparing the base case and VCE scenario. A median saving of €80 (95% CI: -1148 to 1284) per patient per year is achieved using VCE (55.1% of simulated patients returned cost savings). Results from the one-way sensitivity analysis (**Figure S1**), showed that the total cost differential between the SOC and VCE was primarily influenced by the costs associated with surgery and the VCE procedure itself. Alterations in the expenses related to medication and fistula repair also resulted in significant fluctuations in the cost difference. In contrast, costs associated with ileoscopy (ILE), magnetic resonance enterography (MRE), computed tomography (CT), and other complications had a minor effect on the overall cost difference. The influence of other factors not depicted in the figure was negligible.

### Real-World Validation

**Analysis flow:** Out of 300 patient records, 276 were retained after applying the selection criteria. Each record was replicated 100 times to generate the 27 600-patient simulated set (**Figure S2**).

**Figure 3.**
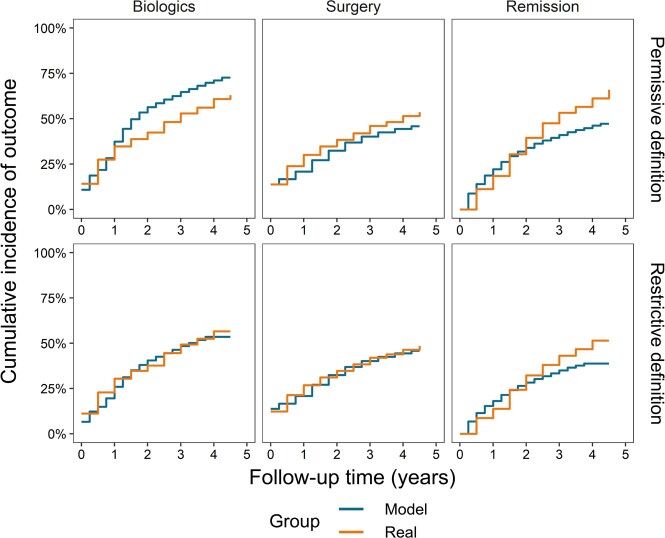
Cumulative Incidence of Outcomes by Permissive and Restrictive Definitions *Top row:* Permissive definition of outcomes, which includes any 6-month period of biologic use, all types of surgeries, and remission lasting any 6 months with no symptoms (CDAI ≤150) preceded by a period with mild, moderate, or severe symptoms (CDAI >150). *Bottom row*: Restrictive definition of outcomes, which includes a period of biologic use of at least 1 year, only elective and emergency surgeries, and remission lasting at least 1 year with no symptoms (CDAI ≤150).

**Cumulative incidence of outcomes:** The first occurrence of an outcome was evaluated using permissive and restrictive definitions (**Table S4**). Overall, predictions align with real-world data (**Figure 3**), but for remission, which is overpredicted for up to 1.5-2.0 years and underpredicted afterward. However, the real data show larger changes over single intervals.

**Figure 4.**
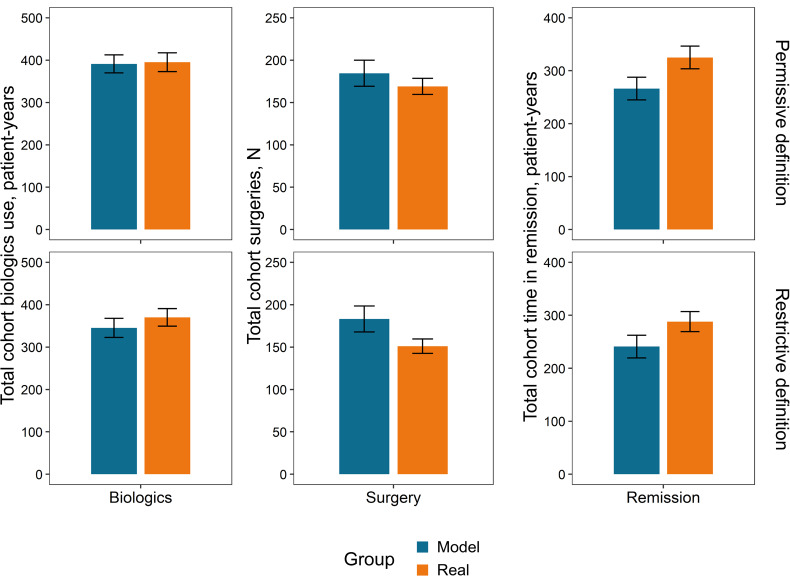
Model vs Real Outcome Totals Across Cohorts Over the Follow-Up Period Outcome totals over the 4.5-year follow-up period are shown according to a permissive definition (top) for biologics (any period of 6 months of use) and surgeries (all surgical procedures). The total time in remission was defined restrictively (bottom) as a minimum of 1-year duration for any period in remission.

**Outcome totals over the follow-up period**: The disease burden over the follow-up period can be quantified as the aggregate resource utilization (biologics and surgery) and course of disease (duration of remission). **Figure 4** compares cumulative outcomes between the real-world and the simulated sets of patients under permissive or restrictive conditions.

### Bayesian Validation

The model’s quantitative validation was based on the probabilities of a hypothetical decision maker accepting the model’s results as representative of the actual results,^31^ with a baseline acceptance rate of 50% (**Table 2**).

**Table 2.** Model Validity Results According to Bayesian Analysis

**Outcome**	**Proximity**	**Permissive (95% CI)**	**Restrictive**
Biologics	True value	80.4% (72.2%-87.5%)	34.3% (25.5%-43.8%)
	5%	99.0% (96.4%-100.0%)	67.6% (58.3%-76.3%)
	10%	99.0% (96.4%-100.0%)	87.3% (80.2%-93.0%)
	20%	99.0% (96.4%-100.0%)	99.0% (96.4%-100.0%)
Surgery	True value	43.1% (33.7%-52.8%)	5.9% (2.2%-11.2%)
	5%	53.9% (44.2%-63.5%)	14.7% (8.6%-22.2%)
	10%	73.5% (64.6%-81.6%)	46.1% (36.5%-55.8%)
	20%	99.0% (96.4%-100.0%)	64.7% (55.2%-73.6%)
Remission	True value	1.0% (0.0%-3.6%)	1.0% (0.0%-3.6%)
	5%	1.0% (0.0%-3.6%)	1.0% (0.0%-3.6%)
	10%	1.0% (0.0%-3.6%)	1.0% (0.0%-3.6%)
	20%	11.8% (6.3%-18.7%)	20.6% (13.4%-28.9%)

For each of the 3 outcomes, the results indicate the probability that a hypothetical decision maker would accept the model’s results as representative of reality according to the indicated parameters. The “true value” indicates that the model result falls within the uncertainty range of the real value. Other percentages indicate the degree of expansion (ie, the proximity of 10% means that the model value is within 10% of the real value, including its uncertainty range).

Abbreviation: CI, confidence interval.

The model’s validity varied primarily based on the outcome and the employed definition. Under the permissive definition, the model reproduces empirical observation accurately in 80.4% of iterations (99% with more permissive proximity) while limiting to 66.7% and 87% accuracy at 5% and 10% proximity allowance under the stringent definition. The model is comparatively less efficient at predicting surgery under either permissive or restrictive criteria (**Table S4**), achieving satisfactory results only within a more permissive proximity range (73.5% at ≥10% proximity).

The model did not predict total time in remission according to either definition. Maximum validity values reached only 11.8% and 20.6% for permissive and restrictive values, respectively, when considering a threshold of coming within 20% of the real total time in remission.

## DISCUSSION

A model for VCE in monitoring CD patients^22,23^ was adapted to Italian specifics and validated in its accuracy and generalizability. The model proved solid in capturing the complexities of CD, except for remission, a multifaceted and nuanced concept that poses significant challenges in clinical definition and modeling. This may be attributed to using the CDAI as the sole criterion for defining remission, which serves as a proxy for inflammatory activity and mucosal healing but does not fully describe the overall course of the disease. The scant empirical longitudinal data available on the evolution of CDAI further limits the model’s predictive accuracy of remission. Exploring larger and multicentric real-world data sets can offer potential avenues for enhancing the model’s predictive performance.

Analyzing the modeled outcomes revealed a steadier increment than the real-world data. This discrepancy may be attributed to the large number of model patients utilized to mitigate the effects of uncertainty in the model outcomes. In contrast, the limited real-world data set may have resulted in more significant fluctuations in cumulative incidence. Additionally, the dynamic nature of the clinical practice, which may involve treatment modifications not accounted for in the model, may have contributed to the observed difference. The model operates within a predetermined treatment framework based on diagnosed pathology and CDAI scores, while physicians may consider factors such as patient preferences and prior treatment history in their decision-making process.

The budget impact analysis appraised potential savings in the mid to long term at around €67 per patient per year, subject to significant uncertainty at sensitivity analysis. Also, the cost estimation presents a challenging interpretation due to the limited availability of data on hospital and healthcare resource utilization and costs in the patient sample analyzed. Nevertheless, the cost estimates are coherent with other European studies.^32–34^ The development of cost differences over time is consistent with previous studies investigating changes in patient management following diagnosis with VCE.^35^

In conclusion, the benefits to CD patients from the capability of VCE to provide a comprehensive assessment of the entire intestinal tract, thereby reducing the necessity for multiple examinations and improving mid- to long-term management, sum up economic considerations into the overall value proposition of the technology. The budget impact analysis indicates that implementing VCE monitoring in patients diagnosed with CD with small bowel involvement would likely increase healthcare expenditure in the first 2 years. However, cost savings are expected in the mid to long term within the specifics of Italian healthcare.

### Limitations

The model is subject to limitations in its attempt to replicate clinical practice programmatically. It evolves through an idealized, predefined treatment framework, does not account for clinician discretionary judgment or clinical inaccuracy, and may not adequately cover the full range of possible CD cases. The real-world validation itself can suffer from the relatively limited statistics of cases in the monocentric cohort of 276 patients, missing data points, or inconsistent data recorded over two decades. Furthermore, when considering shorter time horizons, the likelihood of agreement between model results and tangible outcomes increases, potentially increasing the certainty in actions taken based on model predictions. Future comprehensive analyses may investigate the additional challenges associated with predicting patient remission. However, despite these limitations, the agreement between the model and real-world results across multiple outcomes is unlikely to be solely attributed to chance.

### Disclosures

R.S., J.D., and R.T.T. are Coreva Scientific GmbH & Co KG employees, which received consultancy fees from Covidien, now part of Medtronic, for executing, analyzing, and disseminating the work presented in this manuscript. C.C. and D.G. received compensation from Coreva Scientific.

## Supplementary Material

Online Supplementary Material
