# Supplementary material for: Validation of a Model Estimating the Budget Impact of Video Capsule Endoscopy for Surveillance of Crohn’s Disease in an Italian Center
Source: J Health Econ Outcomes Res. 2024 Mar 7;11(1):67–74. doi: 10.36469/001c.92880 (PMC10924452; doi:10.36469/001c.92880)
Supplement: Online Supplementary Material [file jheor_2024_11_1_92880_199123.pdf]

Journal of Health Economics and Outcomes Research

Online Supplementary Material

Validation of a Model Estimating the Budget Impact of Video Capsule Endoscopy for Surveillance of Crohn's Disease in an Italian Center. *JHEOR*. 2024;11(1):67-74. doi:10.36469/jheor.2024.92880

Table S1: Markov Model Transition Probability

Table S2: Crohn's Disease Location Transitions

Table S3: Cost Inputs

Table S4: Permissive and Restrictive Outcome Definitions

Figure S1: One-Way Sensitivity Analysis

Figure S2: Flow Plan of the Analysis

This supplementary material has been provided by the authors to give readers additional information about their work.

This is an open-access article distributed under the terms of the Creative Commons Attribution 4.0 International License (CCBY-4.0). View this license's legal deed at http://creativecommons.org/licenses/by/4.0 and legal code at http://creativecommons.org/licenses/by/4.0/legalcode for more information.

Table S1. Markov Model Transition Probability

**Markov Model States**	**Undiagnosed CD**	**Remission**	**Non-Active Symptomatic**	**Active Symptomatic**	**Active Nonsymptomatic**	**GI Hemorrhage**	**Treatment**	**Treatment Failure**	**Surgery**	**Post-surgery**	**Death**
Undiagnosed CD	0.725660555	0	0	0	0.011904762	0	0.2420711	0	0.020363614	0	0
Remission	0	0.823549	0.058816991	0.058816991	0.058816991	0	0	0	0	0	0
Non-active symptomatic	0	0.1647927	0.534319235	0.058816991	0	0	0.2420711	0	0	0	0
Active symptomatic	0	0.1647927	0.013678085	0.379475333	0.164792705	0.01482649	0.2420711	0	0.020363614	0	0
Active non-symptomatic	0	0.1647927	0	0.164792705	0.623319724	0.01482649	0.0119048	0	0.020363614	0	0
GI hemorrhage	0	0	0	0	0	0	0.8	0	0.2	0	0
Treatment	0	0.0599244	0	0	0	0	0.7680927	0.171982879	0	0	0
Treatment failure	0	0	0	0	0	0	0.9796364	0	0.020363614	0	0
Surgery	0	0	0	0	0	0	0	0	0	1	0
Post-surgery	0	0.127316	0	0	0.07884413	0	0.6453102	0	0.148529659	0	0
Death	0	0	0	0	0	0	0	0	0	0	1

Abbreviations: CD, Crohn's disease; GI, gastrointestinal.

Table S2. Crohn's Disease Location Transitions

Location Transitions	L1	L2	L3	L4
L1	0.963333235	0.0122223	0.012222255	0.012222255
L2	0	0.98890807	0.005545966	0.005545966
L3	0	0	0.996986405	0.003013595
L4	0	0	0	1

Table S3. Cost Inputs

Item	Costs € (DRG code)
Ileocolonoscopy, per procedure	74.00 (45.23)[28]
VCE, per procedure	850.00 (45.13.1)[28]
Patency capsule, per procedure	133.83[29]
MRE, per procedure	160.10 (88.95.4)[28]
Fecal calprotectin test, per procedure	12.05 (90.12.A Veneto)[30]
CRP test	5.00 (90.72.3)[28]
Infliximab, per year	12 254.40[a]
Infliximab, administration cost	10.65 (99.25)[28]
Adalimumab, per year	10 197.29[a]
Adalimumab, administration cost	8.50 (99.29.5)[1]
Vedolizumab, per year	15 349.06[a]
Vedolizumab, administration cost	10.65 (99.25)[1]
5-ASA, per year	10.96[a]
5-ASA, administration cost	–
Corticosteroids, per year	29.22[a]
Corticosteroids, administration cost	–
Azathioprine, per year	255.68[a]
Azathioprine, administration cost	–
Open bowel surgery	7611.36[2]
Abscess drainage	4812.69 (171)[2]
Fistula repair	5514.36 (156)[2]
Stricturing repair	4805.04 (153)[2]
Capsule retention	914.83 (189)[2]
Bowel perforation	914.83 (189) [2]

Abbreviations: ASA, 5-aminosalicylic acid; CRP, C-reactive protein; MRE, magnetic resonance enterography; VCE, video capsule endoscopy.
[a]Biologics and conventional annual costs from 2023 wholesale prices.

REFERENCES

1. Regione Emilia Romagna. PRESTAZIONI DI ASSISTENZA SPECIALISTICA AMBULATORIALE, ottobre 2022. Accessed September 19, 2023. https://salute.regione.emilia-romagna.it/ssr/strumenti-e-informazioni/nomenclatore-tariffario-rer/ottobre-2022
2. Regione Emilian Romagna. DRG 2023 - 878.

Table S4. Permissive and Restrictive Outcome Definitions

		Biologics	Surgery	Remission
Permissive	Model	Continuous 6-month use (2 consecutive intervals)	Keep all (fistulectomy, emergency, elective)	Consecutive 6-month period CDAI ≤150
	RWD	Continuous 6-month use (1 interval)	All non-missing	Any 6 months CDAI ≤ 150
Restrictive	Model	Minimum 1-year continuous use (4 consecutive intervals)	Keep only elective and emergency surgery (state "i")	Consecutive 1-year period CDAI ≤150
	RWD	Minimum 1-year continuous use (2 consecutive intervals)	Drop fistulectomies (keep only "yes")	Consecutive 1-year period CDAI ≤150

Abbreviation: RWD, real-world data.

Figure S1. One-Way Sensitivity Analysis

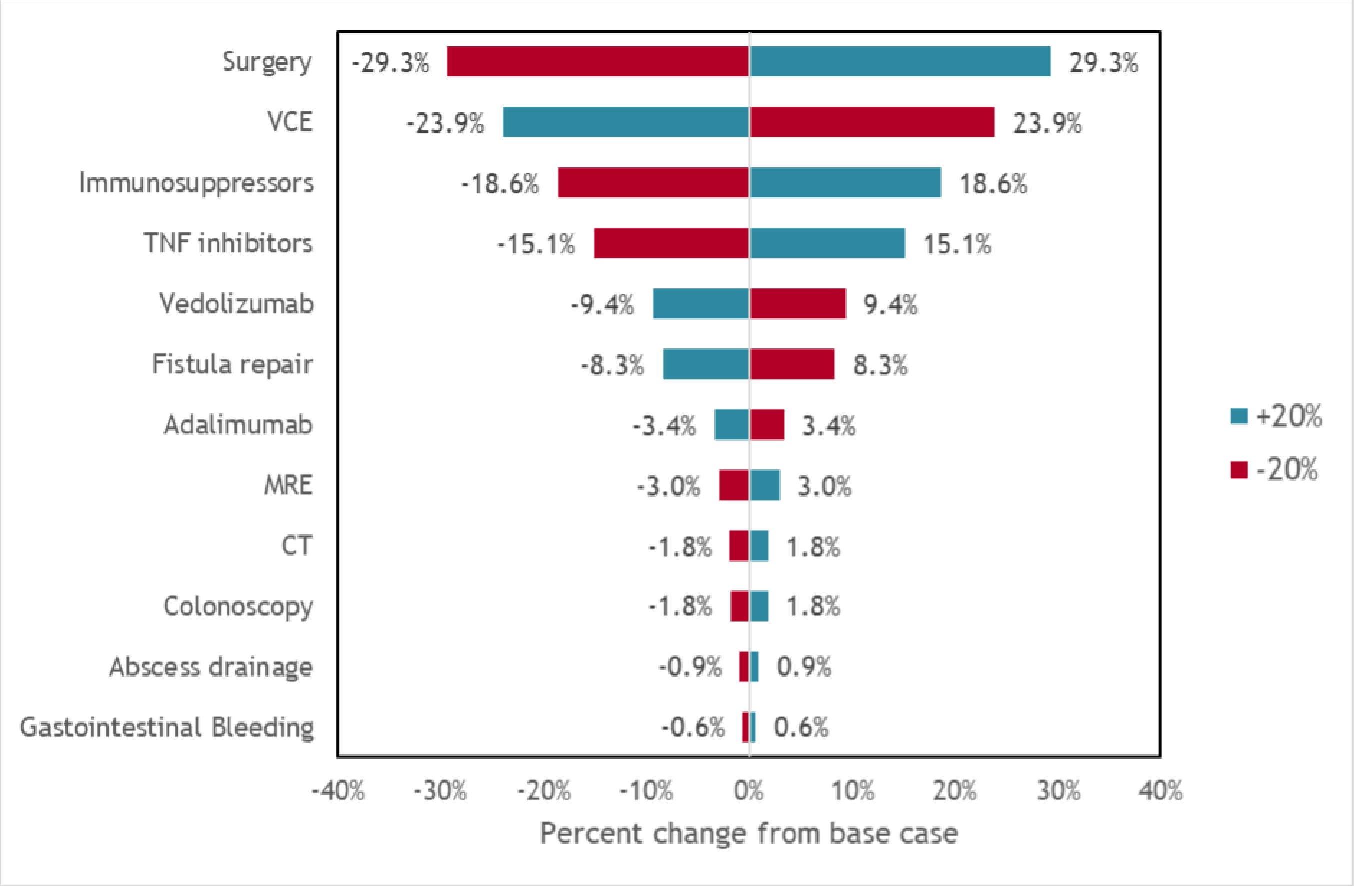

This figure presents the impact of a ±20% variation in relevant cost inputs on the base case results (5-year total cost difference between standard of care and VCE). Positive values favor VCE.
Abbreviations: CT, computer tomography; MRE, magnetic resonance enterography; VCE, video capsule endoscopy.

Figure S2. Flow Plan of the Analysis

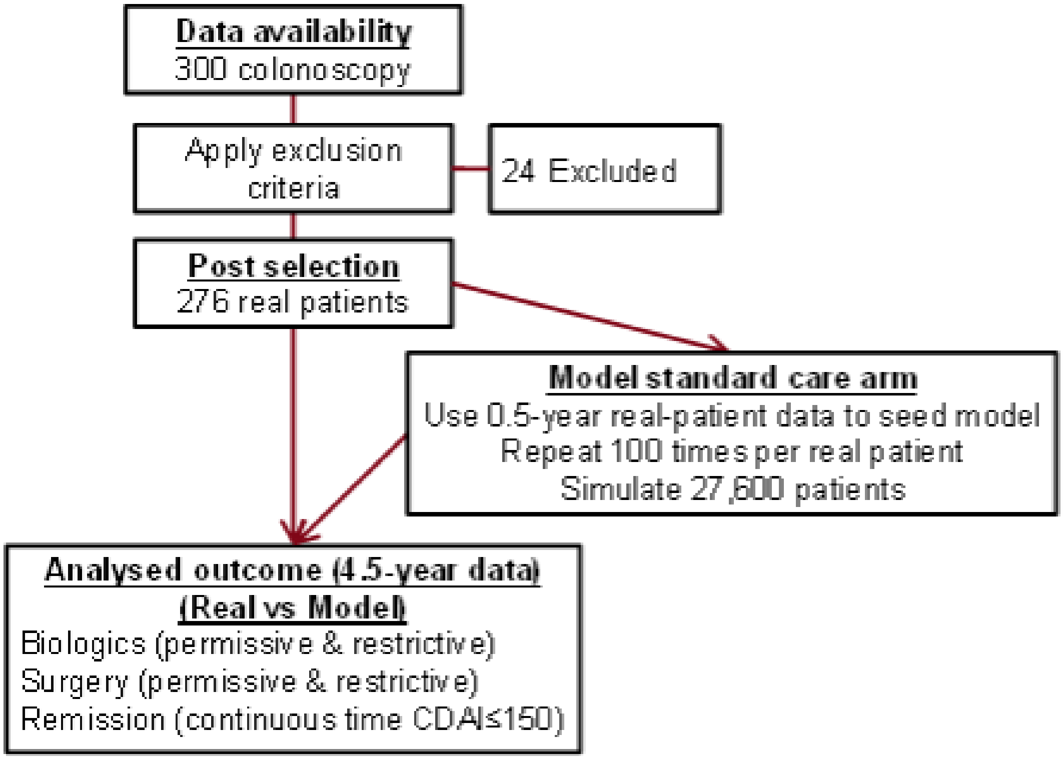

Flow plan shows analysis from acquiring pseudonymized real patient data to comparing outcomes with model-generated data on patients after applying selection criteria.
